# Coenzyme Q10 Supplementation Modulates Hepatic Lipidomic Alterations and Attenuates Metabolic Dysfunction-Associated Steatohepatitis in Mice

**DOI:** 10.3390/nu18040588

**Published:** 2026-02-11

**Authors:** Yula Go, Heeju Joung, Sang Yun Han, Jayong Chung

**Affiliations:** 1 Department of Food & Nutrition, Kyung Hee University, Seoul 02447, Republic of Korea; 2 Department of Chemistry, Gachon University, Seongnam 13120, Republic of Korea

**Keywords:** metabolic dysfunction-associated steatohepatitis, coenzyme Q10, lipidomics, phospholipids, sphingolipids, liver

## Abstract

Background/Objectives: Metabolic dysfunction-associated steatohepatitis (MASH) is a chronic liver disorder with limited effective therapeutic options. Emerging lipidomic studies suggest that alterations in membrane-associated lipids contribute to MASH pathophysiology; however, nutritional interventions capable of modifying these lipid alterations remain poorly defined. This study aimed to investigate the effects of coenzyme Q10 (CoQ) supplementation on hepatic lipidomic remodeling in a methionine- and choline-deficient (MCD) diet-induced mouse model of MASH. Methods: Male C57BL/6J mice were fed a methionine- and choline-sufficient diet or an MCD diet for 4 weeks, with MCD-fed mice receiving vehicle or CoQ (100 mg/kg body weight/day). Hepatic lipid profiles were assessed using untargeted LC–MS-based lipidomics, and expression of genes involved in phospholipid and sphingolipid metabolism was quantified by quantitative real-time PCR. Results: CoQ supplementation significantly attenuated liver injury induced by the MCD diet, as evidenced by reduced histological severity and decreased serum ALT and AST levels. Lipidomic analyses revealed marked alterations in hepatic phospholipid and sphingolipid profiles during MASH development. CoQ was associated with remodeling of phospholipid composition, increasing phosphatidylcholine (PC) species and reducing phosphatidylethanolamine (PE) species, resulting in an increased hepatic PC to PE ratio. This change was accompanied by upregulation of *Pemt* (phosphatidylethanolamine N-methyltransferase). In contrast, sphingolipid accumulation induced by the MCD diet remained largely unchanged by CoQ, and *Smpd1* (sphingomyelin phosphodiesterase 1) expression was not altered. Conclusions: CoQ supplementation was associated with attenuation of MCD diet-induced MASH and modulation of hepatic phospholipid homeostasis, supporting its potential as a nutritional intervention targeting membrane lipid dysregulation in MASH.

## 1. Introduction

Metabolic dysfunction-associated steatohepatitis (MASH), formerly known as nonalcoholic steatohepatitis, is a progressive form of metabolic dysfunction-associated steatotic liver disease (MASLD) characterized by hepatic steatosis accompanied by inflammation and hepatocellular injury, and can progress to fibrosis, cirrhosis, and hepatocellular carcinoma [1]. Despite its increasing prevalence and clinical significance, effective pharmacological and dietary interventions for MASH remain limited. Accumulating evidence indicates that dysregulation of hepatic lipid metabolism is critical to MASH pathogenesis [2,3]; however, the specific lipid classes and molecular mechanisms underlying disease progression and therapeutic response are not yet fully understood.

While triglyceride accumulation is a defining feature of steatosis, recent lipidomic studies have demonstrated that MASH is associated with broad alterations in membrane-associated lipid classes, including phospholipids and sphingolipids [4,5,6]. These lipid classes constitute essential components of cellular membranes and are involved in the regulation of membrane physical properties as well as intracellular signaling processes. Alterations in phospholipid composition, particularly imbalances between phosphatidylcholine (PC) and phosphatidylethanolamine (PE), have been linked to impaired membrane integrity, defective lipoprotein assembly, and increased susceptibility to hepatocellular stress [7,8]. Similarly, accumulation of bioactive sphingolipids such as ceramides and sphingomyelins has been implicated in inflammation, insulin resistance, and hepatocellular injury in MASH [9,10,11]. Together, these findings suggest that membrane lipid remodeling represents an important but incompletely characterized aspect of MASH pathophysiology. Critically, nutritional interventions that can effectively modify these lipid alterations remain unexplored and are therefore needed.

Coenzyme Q10 (CoQ) is a lipid-soluble molecule essential for mitochondrial electron transfer and cellular redox homeostasis. It has been reported to exert hepatoprotective effects in various models of liver injury [12,13,14,15]. Beyond its well-known roles in mitigating oxidative stress and improving mitochondrial function, emerging evidence suggests that CoQ may influence lipid metabolism [16,17]. However, the effects of CoQ supplementation on hepatic lipidomic remodeling, particularly membrane-associated phospholipids and sphingolipids, have not been examined in MASH.

Although high-fat diet-induced obesity represents the major clinical drivers of MASH, the methionine- and choline-deficient (MCD) diet model is widely used to investigate hepatic lipid remodeling [6,18]. It reliably reproduces key histopathological features of MASH in humans and induces a more severe hepatic phenotype within a relatively short experimental phenotype. Accordingly, the MCD model was used in the present study.

Therefore, the aim of the present study was to investigate the effects of CoQ supplementation on MCD diet-induced MASH, with a particular focus on comprehensive hepatic lipidomic alterations. Using untargeted lipidomics combined with gene expression analyses, we sought to characterize changes in phospholipid and sphingolipid composition during MASH development and to determine how CoQ supplementation modulates these lipid alterations.

## 2. Materials and Methods

### 2.1. Animals and Diets

Male C57BL/6J mice were obtained from Central Lab. Animal Inc. (Seoul, Republic of Korea) and raised in a controlled environment at 20 ± 2 °C with humidity of 50 ± 10%, and a 12 h dark–light cycle. All mice had free access to drinking water and diet during the experimental periods. The procedures were approved by the Kyung Hee University Institutional Animal Care and Use Committee (KHUIACUC) (KHSASP-22-116). Sample size was determined based on our previous study using the same MCD-induced MASH model [15]. Following a 1-week acclimation period, the mice were randomized into three groups (n = 6~9/group) according to their body weights. One group was fed the methionine- and choline-sufficient (MCS) diet (A02082003BY, Research diets Inc., New Brunswick, NJ, USA). The other two groups were fed a methionine- and choline-deficient (MCD) diet (A02082002BR, Research diets Inc.). According to the manufacturer’s specifications, the MCS diet contained 3 g methionine and 2 g choline bitartrate per kg of diet, whereas the MCD diet contained no added methionine or choline. All groups received daily oral gavage of the same volume of vehicle (corn oil). Among the MCD-fed mice, one group received vehicle (corn oil) alone, whereas the other group received CoQ (100 mg/kg body weight; Acros Organics, Geel, Belgium) dissolved in corn oil by oral gavage once daily. Because CoQ is a lipid-soluble antioxidant, corn oil was used as the vehicle to enhance bioavailability [19,20]. After 4 weeks of treatment, the mice were anesthetized using carbon dioxide. Blood samples were collected via cardiac puncture. Liver tissues were excised and fixed in 10% neutral buffered formalin or snap-frozen in liquid nitrogen.

### 2.2. Biochemical Parameters and Histopathological Analyses

Aspartate aminotransferase (AST) and alanine aminotransferase (ALT) activities in serum were measured using specific diagnostic kits (Asan Pharmaceutical, Hwasung, Republic of Korea).

Paraffin-embedded liver sections were sliced into 5 μm thick sections and stained with hematoxylin and eosin (H&E). Histological sections were observed under an optical microscope (Olympus, Tokyo, Japan) at ×400 magnification. The degree of MASH was estimated by examining steatosis, inflammation, and hepatocyte ballooning. The degree of steatosis (0–3), hepatocellular ballooning (0–2), and lobular inflammation (0–3) were summed to calculate the MASLD activity score (MAS). The MAS evaluation was conducted by investigators blinded to the experimental group assignments.

### 2.3. Lipidomic Anlaysis

Tissue (15 mg) was mixed with 270 μL of methanol and 10 μL of internal standard (SPLASH^®^ LipidoMIX^®^ internal standard, Avanti Polar Lipids, Inc., Alabaster, AL, USA). The mixture was homogenized three times for 10 s using a tissue tearor (Biospec Inc., Bartlesville, OK, USA), followed by sonication in an ice bath for 5 min. The homogenized sample was incubated at −20 °C for 1 h to precipitate proteins. Subsequently, 900 μL of cold methyl *tert*-butyl ether (MTBE) was added, and the solution was vortexed for 30 s at room temperature. After that, 315 μL of cold water was added, and the mixture was vortexed again for 30 s at room temperature. The sample was then centrifuged at 14,000 rpm for 20 min at 4 °C. The separated upper layer was transferred and dried using a centrifugal evaporator (EYELA, Tokyo, Japan) at 25 °C. The dried residue was reconstituted into 100 μL of isopropanol/acetonitrile (1:1, *v*/*v*) solution. After centrifuging at 15,000 rpm for 20 min at 4 °C, the upper layer was collected for further analysis.

Lipidomic profiling of mouse liver tissue was performed using liquid chromatography–mass spectrometry (LC-MS) by electrospray ionization source (ESI) in positive ion mode. Reverse-phase chromatography was performed on a C18 column (1.7 μm, 2.1 mm × 100 mm), and an Orbitrap Exploris 120 (Thermo Fisher Scientific Co., Waltham, MA, USA) was utilized for mass spectrometry. Data acquisition was conducted in the *m*/*z* range of 103–1500 with high resolution. Lipidomic sample preparation and LC-MS analyses were performed in a randomized order under batch-controlled conditions.

Untargeted lipidomic data were processed using Compound Discoverer 3.3 software (Thermo Fisher Scientific Co.). Lipid annotation was performed by matching spectral data against mzCloud library, LipidBlast in silico library(ddMS2), ChemSpider, and LIPID MAPS^®^ databases. Lipid features were grouped and filtered based on accurate mass (±5 ppm), retention time alignment, signal-to-noise ratio, and presence in biological samples versus blank. As a result, only lipid annotations that were reliably selected from the Compound Discoverer results were utilized for data analysis. Lipid quantification was performed with the relevant internal standards and log-transformed prior to statistical analyses.

### 2.4. Quantitative Real-Time RT-PCR

Total RNA from liver tissue was isolated using the RNeasy Mini Kit (Qiagen, Hilden, Germany) following the manufacturer’s instructions. The concentration and integrity of isolated RNA were assessed using a NanoDrop^TM^ spectrophotometer (Thermo Fisher Scientific Co.) and agarose gel electrophoresis. A total of 500 ng of RNA was reverse transcribed into cDNA using the PrimeScript^TM^ RT reagent kit (Takara Bio Inc., Shiga, Japan). Real-time PCR was then performed using the TB Green Premix Ex Taq II kit (Takara Bio Inc.) under the following thermal cycling conditions: an initial denaturation step at 95 °C for 30 s, followed by 40 cycles of denaturation at 95 °C for 5 s and annealing/extension at 60 °C for 30 s. The primer sequences are listed in Supplementary Table S1. The 2^−ΔΔCt^ method was employed to calculate the relative expression of the target genes, which was then normalized to the expression of the *Gapdh* housekeeping gene.

### 2.5. Statistical Analyses

Statistical analysis was conducted using SAS 9.4. All data were presented as means ± SEM. Normality of data distribution was confirmed using the Shapiro–Wilk test. Differences among groups were compared by one-way ANOVA followed by Duncan’s post hoc test. A *p* value < 0.05 was considered statistically significant.

Statistical analysis of lipidomic data was conducted with MetaboAnalyst 6.0 (www.metaboanalyst.ca). For lipidomic analyses, statistical significance was determined using false discovery rate (FDR)-adjusted *p* values to control for multiple comparisons, and *p* < 0.05 was considered statistically significant. In addition, a hierarchical analytical strategy was applied. Global lipidomic patterns were first assessed using multivariate analysis, including sparse partial least squares–discriminant analysis (sPLS-DA), to evaluate overall group separation. Subsequently, lipid species were classified according to lipid class and subclass, and statistical testing was performed within these predefined biological categories.

## 3. Results

### 3.1. CoQ Decreases the Severity of MASH Induced by an MCD Diet in Mice

**Figure 1:**
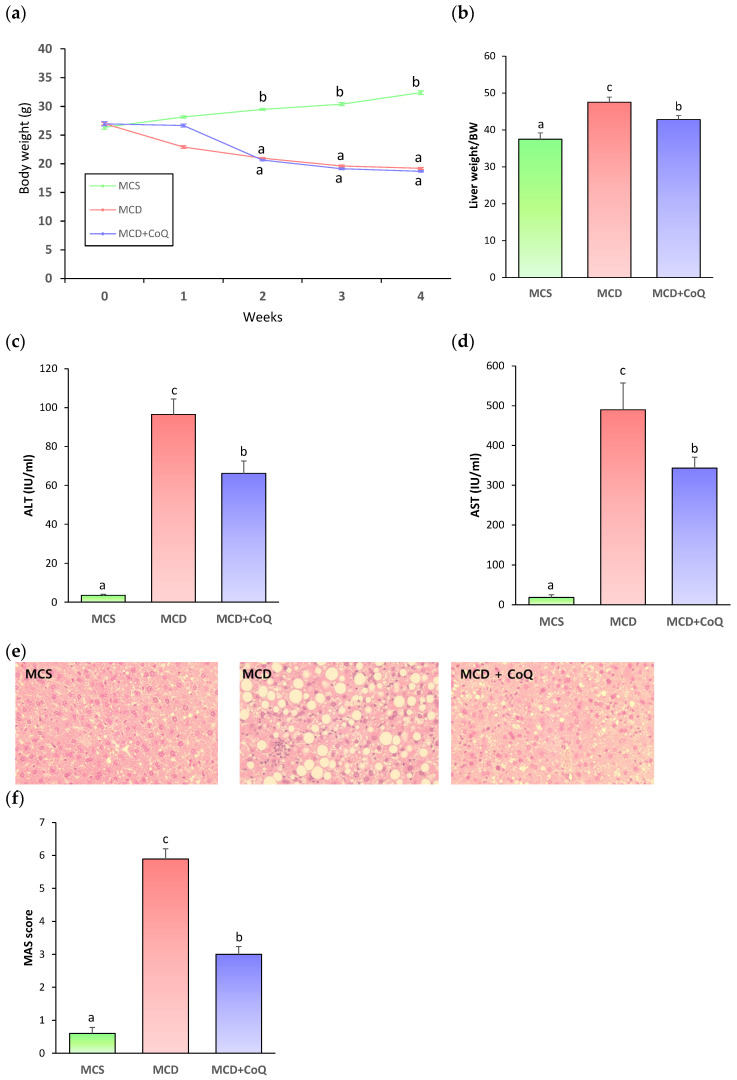
CoQ supplementation attenuates MCD diet-induced liver injury and histopathological features of MASH in mice. (**a**) Body weight changes during the 4-week feeding period in mice fed a methionine- and choline-sufficient (MCS) diet with vehicle (corn oil), a methionine- and choline-deficient (MCD) diet with vehicle (corn oil), or an MCD diet supplemented with CoQ dissolved in corn oil (MCD + CoQ). The 0 time point indicates the start of dietary intervention. Different superscript letters indicate statistically significant differences (*p* < 0.05) at weeks 2, 3 and 4. (**b**) Liver index expressed as the ratio of liver weight (mg) to body weight (g) in each experimental group. (**c**,**d**) Serum alanine aminotransferase (ALT) and aspartate aminotransferase (AST) levels. (**e**) Representative H&E-stained liver sections showing hepatic steatosis, lobular inflammation, and hepatocellular ballooning (×400 magnification). (**f**) Quantitative evaluation of the metabolic dysfunction-associated steatotic liver disease activity score (MAS) in each group. Data are presented as mean ± SEM (n = 6~9 per group). Different superscript letters indicate statistically significant differences (*p* < 0.05).

To evaluate whether CoQ supplementation attenuates MCD diet-induced MASH, C57BL/6J mice were fed an MCD diet with vehicle (MCD group) or CoQ (MCD + CoQ group) for 4 weeks, while control mice were fed an MCS diet (MCS group). At the end of the feeding period, body weight was significantly lower in the MCD group than in the MCS group; however, no significant difference was observed between the MCD and MCD + CoQ groups (Figure 1a).

The liver index, defined as the ratio of liver weight to body weight, was significantly increased in the MCD group compared with the MCS group, whereas CoQ supplementation significantly reduced the liver index in the MCD + CoQ group relative to the MCD group (Figure 1b). In line with these findings, the MCD diet induced pronounced liver injury, as indicated by significantly elevated serum ALT and AST levels compared with the MCS group. CoQ supplementation significantly reduced serum ALT and AST levels by approximately 30% compared with the MCD group (Figure 1c,d).

Histopathological examination revealed severe hepatic steatosis, lobular inflammation, and hepatocellular ballooning in the MCD group (Figure 1e). In contrast, CoQ supplementation markedly reduced both the area and size of lipid droplets and alleviated inflammatory cell infiltration and ballooning degeneration. Quantitative MAS evaluation further confirmed these observations, with the mean MAS score exceeding 5 in the MCD group, indicating established MASH, whereas the MAS score in the MCD + CoQ group was reduced by approximately 50% compared with the MCD group (Figure 1f).

### 3.2. Alterations in Hepatic Lipidomic Profiles in MASH and Effects of CoQ Supplementation

To characterize MASH-associated alterations in hepatic lipidomic profiles and to determine the effects of CoQ supplementation, comprehensive untargeted lipidomic analyses were performed in liver tissues from the MCS, MCD, and MCD + CoQ groups. Across all groups, a total of 424 phospholipid species and 102 sphingolipid species were identified. Major phospholipid subclasses included PC, PE, phosphatidic acid (PA), phosphatidylglycerol (PG), phosphatidylserine (PS), and phosphatidylinositol (PI), with PC and PE accounting for the largest number of identified species. Major sphingolipid subclasses comprised ceramide, sphingomyelin, and glycosphingolipids, with ceramide and sphingomyelin representing the majority of detected sphingolipid species. A complete list of lipid species and subclass classifications is provided in Supplementary Table S2.

**Figure 2:**
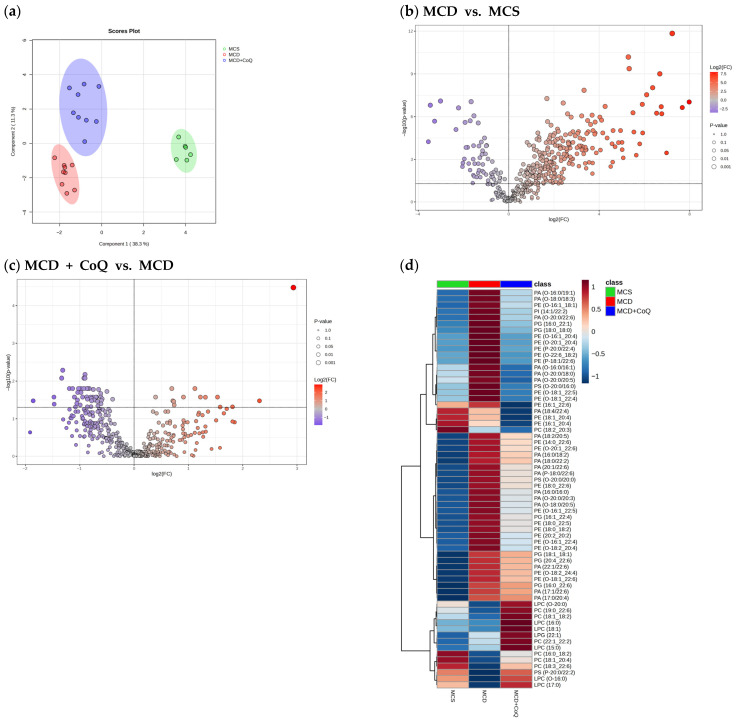
Alterations in hepatic phospholipid profiles induced by the MCD diet and modulated by CoQ supplementation. (**a**) sPLS-DA score plot showing separation of hepatic phospholipid profiles among the MCS, MCD, and MCD + CoQ groups. (**b**) Volcano plot illustrating differential hepatic phospholipid species between the MCD and MCS groups. (**c**) Volcano plot illustrating differential hepatic phospholipid species between the MCD + CoQ and MCD groups. (**d**) Hierarchical heatmap clustering analysis showing significantly altered phospholipid species. False discovery rate (FDR)- adjusted *p* values < 0.05 were used for multiple comparisons of lipid species. MCS: methionine- and choline-sufficient diet-fed group; MCD: methionine- and choline-deficient diet-fed group; MCD + CoQ: methionine- and choline-deficient diet with CoQ supplementation.

Multivariate analysis revealed clear group-specific differences in hepatic lipidomic profiles. The sPLS-DA plot based on phospholipid species demonstrated distinct separation among the MCS, MCD, and MCD + CoQ groups (Figure 2a), indicating marked lipidomic alterations associated with MASH and modulation by CoQ supplementation. Volcano plot analysis comparing the MCD and MCS groups identified extensive changes in hepatic phospholipid species, with 247 species significantly upregulated and 60 species significantly downregulated in the MCD group (Figure 2b). In contrast, volcano plot analysis comparing the MCD + CoQ and MCD groups showed a shift toward downregulation of phospholipid species, with 50 species significantly downregulated and 14 species significantly upregulated following CoQ supplementation (Figure 2c). Hierarchical clustering analysis further confirmed group-specific lipidomic patterns, with samples from the MCD + CoQ group exhibiting abundance profiles distinct from those of the MCD group (Figure 2d).

### 3.3. Effects of CoQ Supplementation on Hepatic Phospholipid Composition in MASH

**Figure 3:**
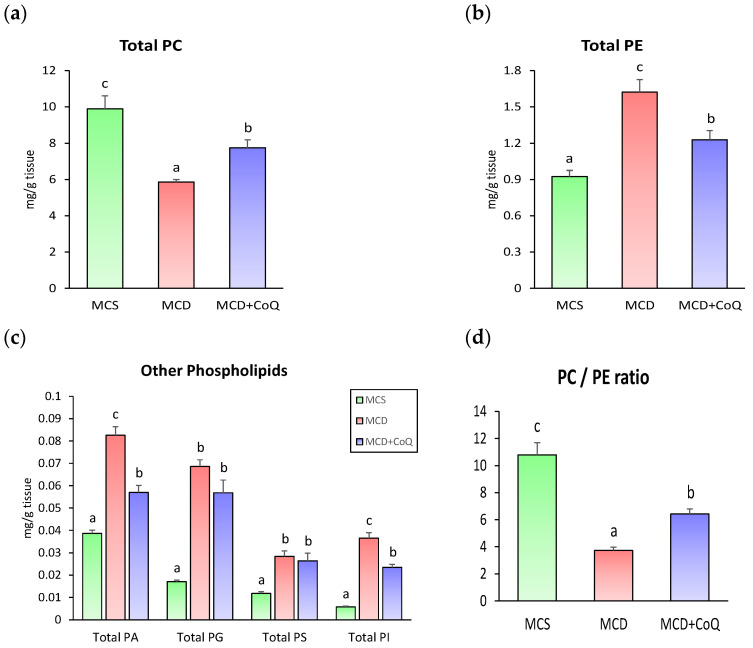
Effects of CoQ supplementation on phospholipid subclass contents and the PC to PE ratio. (**a**) Total phosphatidylcholine (PC) content. (**b**) Total phosphatidylethanolamine (PE) content. (**c**) Total contents of phosphatidic acid (PA), phosphatidylglycerol (PG), phosphatidylserine (PS), and phosphatidylinositol (PI). (**d**) Hepatic PC to PE ratio. Data are presented as mean ± SEM (n = 6~9 per group). Different superscript letters indicate statistically significant differences (*p* < 0.05). MCS: methionine- and choline-sufficient diet-fed group; MCD: methionine- and choline-deficient diet-fed group; MCD + CoQ: methionine- and choline-deficient diet with CoQ supplementation.

To further characterize phospholipid alterations, phospholipid species were grouped into major subclasses. Compared with the MCS group, the MCD group showed a significant reduction in total PC content (Figure 3a), whereas total PE content was significantly increased, reaching approximately 1.8-fold higher levels (Figure 3b). In addition, total levels of PA, PG, PS, and PI were significantly higher in the MCD group than in the MCS group (Figure 3c).

CoQ supplementation significantly modified these MCD-induced phospholipid alterations. In the MCD + CoQ group, total PC content was increased, and total PE content was reduced compared with the MCD group, resulting in a significant increase in the hepatic PC to PE ratio (Figure 3d). Total PA and PI contents were also significantly reduced by CoQ supplementation, whereas PG and PS contents were not significantly altered.

**Figure 4:**
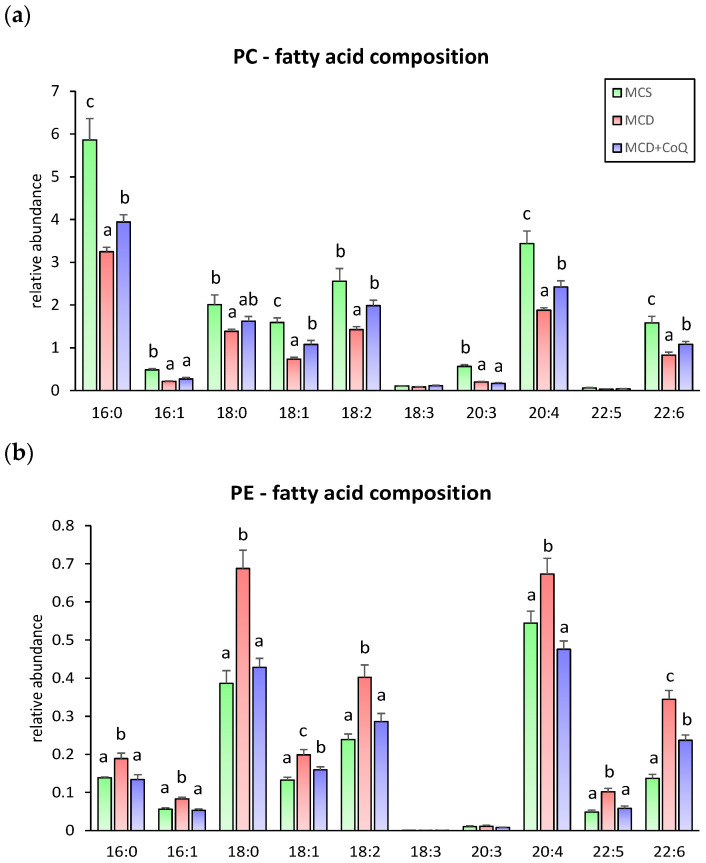
Fatty acid composition of phospholipid species in response to CoQ supplementation. (**a**) Fatty acid composition of phosphatidylcholine (PC) species. (**b**) Fatty acid composition of phosphatidylethanolamine (PE) species. Data are presented as mean ± SEM (n = 6~9 per group). Different superscript letters indicate statistically significant differences (*p* < 0.05). MCS: methionine and choline sufficient diet–fed group; MCD: methionine and choline deficient diet–fed group; MCD + CoQ: methionine and choline deficient diet with CoQ supplementation.

Analysis of fatty acid composition within phospholipid species revealed additional effects of CoQ supplementation. In PC species, most fatty acids were significantly decreased in the MCD group compared with the MCS group (Figure 4a). CoQ supplementation significantly increased the levels of most PC-associated fatty acids, including polyunsaturated fatty acids such as 18:2 and 22:6, restoring them to levels comparable to those in the MCS group. In contrast, fatty acids within PE species were generally increased in the MCD group, and CoQ supplementation reduced these elevated levels, resulting in fatty acid profiles similar to those observed in the MCS group (Figure 4b).

### 3.4. Effects of CoQ Supplementation on Hepatic Sphingolipid Species in MASH

**Figure 5:**
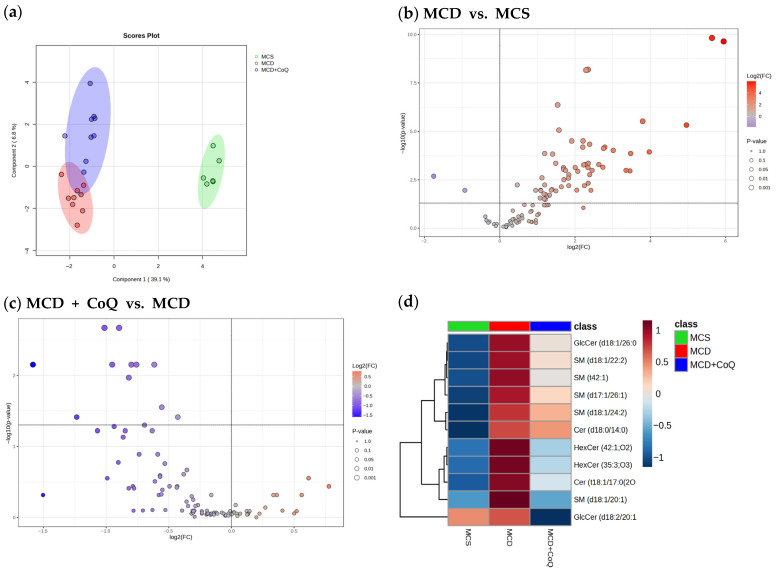
Alterations in hepatic sphingolipid profiles induced by the MCD diet and modulated by CoQ supplementation. (**a**) sPLS-DA score plot of sphingolipid species. (**b**) Volcano plot comparing MCD vs. MCS. (**c**) Volcano plot comparing MCD + CoQ vs. MCD. (**d**) Hierarchical heatmap clustering analysis showing significantly altered sphingolipid species. False discovery rate (FDR)-adjusted *p* values < 0.05 were used for multiple comparisons of lipid species. MCS: methionine- and choline-sufficient diet-fed group; MCD: methionine- and choline-deficient diet-fed group; MCD + CoQ: methionine- and choline-deficient diet with CoQ supplementation.

Multivariate analysis of sphingolipid species showed clear separation between the MCS and MCD groups, whereas partial overlap was observed between the MCD and MCD + CoQ groups (Figure 5a). Volcano plot analysis identified 60 sphingolipid species that were significantly increased in the MCD group compared with the MCS group (Figure 5b). In contrast, 11 sphingolipid species were significantly decreased following CoQ supplementation when compared with the MCD group (Figure 5c). Heatmap analysis highlighted specific sphingolipid species that were altered by CoQ supplementation (Figure 5d).

**Figure 6:**
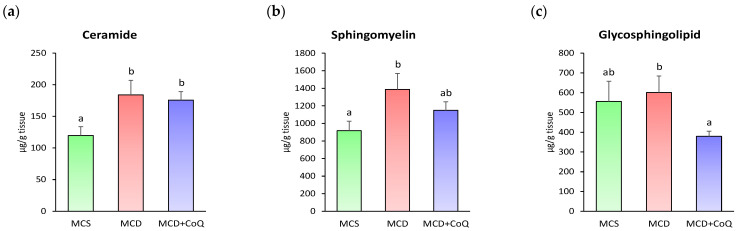
Effects of CoQ supplementation on sphingolipid subclass contents. (**a**) Ceramide. (**b**) Sphingomyelin. (**c**) Glycosphingolipid. Data are presented as mean ± SEM (n = 6~9/group). Different superscript letters indicate significant differences (*p* < 0.05). MCS: methionine- and choline-sufficient diet-fed group; MCD: methionine- and choline-deficient diet-fed group; MCD + CoQ: methionine- and choline-deficient diet with CoQ supplementation.

When sphingolipids were examined at the subclass level, the total contents of ceramide and sphingomyelin were significantly increased in the MCD group relative to the MCS group (Figure 6a,b). However, no significant differences were observed in the total abundance of these sphingolipid subclasses between the MCD and MCD + CoQ groups.

### 3.5. Effects of CoQ Supplementation on Hepatic Expression of Genes Involved in Phospholipid and Sphingolipid Metabolism

To explore molecular mechanisms underlying the observed lipidomic alterations, we analyzed hepatic expression of genes involved in phospholipid and sphingolipid metabolism in MCD-fed MASH mice with or without CoQ supplementation.

**Figure 7:**
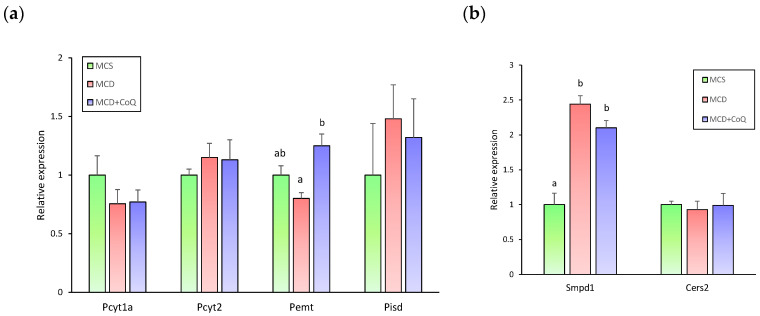
Hepatic expression of genes involved in phospholipid and sphingolipid metabolism. (**a**) mRNA expression of phospholipid metabolism-related genes (*Pemt*, *Pcyt1a*, *Pcyt2*, *Pisd*). (**b**) mRNA expression of sphingolipid metabolism-related genes (*Smpd1*, *Cers2*). Data are presented as mean ± SEM (n = 6~9 per group). Different superscript letters indicate statistically significant differences (*p* < 0.05). MCS: methionine- and choline-sufficient diet-fed group; MCD: methionine- and choline-deficient diet-fed group; MCD + CoQ: methionine- and choline-deficient diet with CoQ supplementation.

Among genes involved in phospholipid metabolism, the expression of *Pemt*, which encodes phosphatidylethanolamine N-methyltransferase and plays a critical role in the conversion of PE to PC, tended to be lower in the MCD group than in the MCS group (*p* = 0.06) (Figure 7a). In contrast, *Pemt* mRNA levels were significantly increased in the MCD + CoQ group compared with the MCD group (Figure 7a). These changes were accompanied by alterations in hepatic phospholipid composition, including a reduced PC to PE ratio in MCD-fed mice and an increased ratio following CoQ supplementation. In contrast, the expression levels of other genes involved in phospholipid synthesis and remodeling were not significantly different among the three groups.

We next examined genes involved in sphingolipid metabolism to assess whether transcriptional changes were associated with sphingolipid alterations observed at the species level. The expression of *Smpd1*, which encodes sphingomyelin phosphodiesterase 1 and catalyzes the hydrolysis of sphingomyelin to ceramide, was significantly upregulated in the MCD group compared with the MCS group (Figure 7b). However, CoQ supplementation did not significantly alter *Smpd1* expression, and this elevated expression was maintained in the MCD + CoQ group. Similarly, the mRNA expression levels of *Cers2* (ceramide synthase 2) did not differ significantly among the MCS, MCD, and MCD + CoQ groups.

## 4. Discussion

In this study, we demonstrate that CoQ supplementation markedly attenuates the MCD diet-induced MASH and is accompanied by specific alterations in hepatic lipid composition. CoQ supplementation was associated with reduced liver injury, as evidenced by improvements in histopathological features and biochemical markers, together with modulation of phospholipid and sphingolipid profiles disrupted by MASH. These findings suggest that changes in hepatic lipid composition represent a characteristic feature of the hepatic response to CoQ supplementation associated with MASH attenuation.

Body weight loss observed in the MCD diet group is a well-recognized characteristic of this model [18,21,22]. Although food intake was not directly measured in the present study, previous studies [18,23] have consistently reported that MCD-fed mice exhibit reduced body weight despite comparable food intake when normalized to body weight, suggesting that body weight loss is not primarily due to decreased caloric intake. Importantly, in the present study, no significant difference in body weight was observed between the MCD and MCD + CoQ groups. Therefore, the protective effects of CoQ supplementation on liver injury observed in this study are independent of MCD diet-induced body weight loss.

Comprehensive lipidomic analyses in the present study revealed profound alterations in hepatic lipid profiles associated with MASH development, extending beyond simple triglyceride accumulation. In particular, we observed extensive changes in phospholipid and sphingolipid species during MCD diet-induced liver injury. Phospholipids and sphingolipids constitute major structural components of cellular membranes and play critical roles in determining membrane biophysical properties and regulating intracellular signaling pathways [24,25]. Consistent with our findings, previous lipidomic studies have reported increased sphingolipid content in MASH models induced by both atherogenic and MCD diets, indicating that alterations in these lipid classes represent common features of MASH pathogenesis [6,26]. Moreover, lipidomic analyses of human liver biopsies from patients with MASH have demonstrated that phospholipids and sphingolipids account for the majority of lipids contributing to the disease-specific lipid signature, whereas triglycerides represent a relatively smaller proportion [27]. Although increased lipid droplet accumulation may influence lipid levels expressed per gram of tissue, the selective and non-uniform alterations observed in membrane-associated lipid classes cannot be attributed to a simple dilution effect caused by lipid vacuole expansion. Further, our analysis revealed that MASH development was characterized by a significant reduction in total PC content accompanied by increases in PE and other phospholipid subclasses, as well as marked elevations in hepatic ceramide and sphingomyelin levels. Together, these findings indicate that dysregulation of membrane lipid composition is one of the characteristic features of hepatic lipidomic alterations observed during MASH development.

In this study, CoQ supplementation was associated with a distinct alteration of hepatic phospholipid profiles at the multivariate level, with the MCD + CoQ group forming a lipidomic pattern clearly distinguishable from that of the untreated MCD group, as demonstrated by sPLS-DA and hierarchical clustering analyses. At the species level, volcano plot analysis showed that several PC species, including PC 18:1_18:2, PC 18:1_20:4, PC 18:3_22:6, and PC 22:1_22:2, were significantly increased, whereas multiple PE species, including PE 18:0_22:5, PE 16:1_20:4, PE 20:2_20:2, PE 16:1_22:6, and PE 18:0_22:6, were significantly decreased following CoQ supplementation. These patterns indicate that although MASH is accompanied by global alterations in phospholipid composition, the effects of CoQ supplementation are selective and species-specific rather than global normalization. In this regard, our findings are in line with a recent multiomics study using the same MCD-induced MASH model [28], in which *Schisandra* lignans extract was shown to ameliorate steatohepatitis primarily by suppressing specific PE species, supporting the concept that targeted modulation of phospholipid species can be sufficient to influence MASH progression.

Although CoQ9 is the predominant endogenous form in rodents, CoQ10 was used in the present study because it is the most commonly used supplemental form. Previous studies [19,20] have demonstrated that exogenously administered CoQ10 increases both CoQ9 and CoQ10 levels in serum, liver, and mitochondria, thereby elevating total CoQ content. Therefore, potential competition between CoQ9 and CoQ10 is unlikely to substantially influence the interpretation of the present findings.

We also found that hepatic PC to PE ratio was significantly decreased in MASH mice and that CoQ supplementation significantly reversed this MASH-associated reduction in the PC to PE ratio. The PC to PE ratio is a well-recognized determinant of cell membrane integrity and has been proposed as a predictor of nonalcoholic liver diseases [29]. Consistent with our findings, a reduced PC to PE ratio has been reported in the livers of MASH mice induced by a high-sugar diet [30] as well as in liver tissues from patients with MASH compared with control livers [7]. Further, genetic deletion of *Pemt* led to a marked decrease in the PC to PE ratio, resulting in loss of membrane integrity and subsequent hepatic injury, including hepatocellular ballooning and progression to steatohepatitis [7]. Such an imbalance between PC and PE has been implicated in altered membrane curvature, impaired lipoprotein assembly, and increased susceptibility to cellular stress in the liver. Therefore, the increase in the PC to PE ratio induced by CoQ supplementation suggests that CoQ may modulate membrane-related properties and thereby contribute to the prevention and/or attenuation of MASH severity.

To explore potential molecular mechanisms underlying the phospholipid alterations observed following CoQ supplementation, we examined the hepatic expression of genes involved in phospholipid metabolism. Among the genes analyzed in the present study, *Pemt* expression tended to be reduced in MASH mice fed the MCD diet and was significantly increased by CoQ supplementation. In contrast, the expression of *Pcyt1a* and *Pcyt2*, which regulate de novo synthesis of PC and PE through the cytidine diphosphate (CDP)-choline and CDP-ethanolamine pathways [31,32], respectively, was not altered by either MCD feeding or CoQ supplementation. Likewise, no significant differences were found in the expression of *Pisd*, which catalyzes the conversion of PS to PE and contributes primarily to mitochondrial PE production, among the three groups. PEMT is predominantly expressed in the liver and localized at endoplasmic reticulum–mitochondria contact sites, where it directly converts PE to PC and serves as a key regulator of the PC to PE ratio, thereby influencing membrane composition and integrity [33]. Importantly, PEMT is the only enzyme capable of directly modulating the PC to PE ratio through phospholipid interconversion rather than through changes in total phospholipid species. Therefore, the selective upregulation of *Pemt* following CoQ supplementation, in the absence of changes in other phospholipid biosynthetic genes, is consistent with the observed increase in the PC to PE ratio and supports a targeted effect of CoQ on phospholipid interconversion rather than global phospholipid synthesis.

Given the localization of PEMT at intracellular membrane interfaces, the observed changes in the PC to PE ratio are most likely reflective of remodeling of intracellular membranes, including mitochondria and mitochondria-associated endoplasmic reticulum membranes, rather than the plasma membrane [34,35]. These membrane systems are particularly sensitive to disturbances in phospholipid metabolism.

Although CoQ is a key component of the mitochondrial electron transport chain, the present study was not designed to directly assess mitochondrial function. Nevertheless, because PEMT-dependent phospholipid remodeling is closely linked to mitochondrial membrane composition, indirect effects of CoQ on mitochondrial homeostasis, oxidative stress, or cellular toxicity cannot be excluded. Future studies investigating the potential secondary contribution of improved mitochondrial stability or activity mediated by CoQ are warranted.

It should be noted that mRNA expression does not always directly reflect enzyme activity. However, previous studies have reported good correlations between the transcript levels of genes examined in this study, including *Pemt*, *Pisd*, *Pcyt2*, and *Smpd1*, and their corresponding protein levels or enzymatic activities [28,36]. Protein-level or enzyme activity measurements, such as PEMT or sphingomyelinase activity, were beyond the scope of this study and represent important directions for future research. In addition, gene expression analysis was limited to selected pathways and may not fully capture the complexity of hepatic lipid metabolism, which should be considered when interpreting the results.

Our study revealed that hepatic ceramide and sphingomyelin species were markedly increased in MASH mice fed the MCD diet. Ceramides and sphingomyelins are important mediators of cellular stress, inflammation, and insulin resistance [9,11,37], and their accumulation has been linked to hepatocellular injury [10,38]. In a study by Gautam et al. [18], however, it was reported that most ceramide species were decreased in the MCD group, with no significant changes observed in sphingomyelin levels. However, an important methodological difference should be considered when interpreting these discrepancies. In the study by Gautam et al. [18], fewer than 30 ceramide and sphingomyelin species were identified, representing a substantially narrower coverage of the hepatic sphingolipidome compared with the 102 sphingolipid species detected in the present study. Supporting our findings, Montandon et al. [6], using both atherogenic and MCD diet-induced models of steatohepatitis, reported enrichment of hepatic sphingolipids, including ceramide and sphingomyelin species, consistent with the pattern observed in our analysis. These observations indicate that sphingolipid perturbations, particularly enrichment of specific ceramide and sphingomyelin species, represent a reproducible characteristic of the MCD model when assessed using comprehensive lipidomic approaches.

Consistent with these findings, multivariate analysis of sphingolipid species in the current study showed a clear separation between the MCD group and the MCS group. On the other hand, the sphingolipidomic profile of the MCD + CoQ group partially overlapped with that of the MCD group. This pattern contrasts with the more pronounced separation observed for phospholipid profiles following CoQ supplementation. Such partial overlap suggests that sphingolipid accumulation represents a relatively stable component of MCD-induced hepatic injury that may be less amenable to modulation by CoQ at the level of global sphingolipid remodeling.

Further, we observed a significant upregulation of *Smpd1* gene expression in the hepatic tissues of MASH mice. *Smpd1* encodes sphingomyelin phosphodiesterase 1, a key enzyme in the salvage pathway, which hydrolyzes sphingomyelin into ceramide, directly contributing to the expansion of the intrahepatic ceramide pool. Previous studies have shown that inhibition of this enzyme reduces the progression of metabolic dysfunction-associated steatotic liver disease [39,40]. In contrast, CoQ supplementation did not significantly alter *Smpd1* gene expression in the present study, which is consistent with our lipidomic findings showing that CoQ did not normalize the elevated total levels of ceramide or sphingomyelin in MASH mice. These observations suggest that CoQ exerts limited effects on the transcriptional regulation of the sphingomyelin–ceramide axis in MASH. Future studies combining CoQ with interventions that directly target *Smpd1* or ceramide metabolism may help to further delineate complementary strategies for modulating sphingolipid dysregulation in MASH. Also, as this study was conducted only in male mice, potential sex-specific differences cannot be excluded, and further studies using female models are warranted.

## 5. Conclusions

In conclusion, the present study demonstrates that CoQ supplementation is associated with attenuation of MCD diet-induced MASH features and is accompanied by selective remodeling of the hepatic lipidome. Our findings highlight that the protective effects of CoQ are associated with phospholipid alterations and an increase in the PC to PE ratio, together with the upregulation of *Pemt*. These findings support the potential use of CoQ as a nutritional strategy targeting membrane lipid dysregulation in MASH.

## Data Availability

The original data obtained in this study are included in the article. Further inquiries can be directed to the corresponding author.

## Supplementary Materials

The following supporting information can be downloaded at: https://www.mdpi.com/article/10.3390/nu18040588/s1, Table S1: Summary of primer sequences. Table S2: Complete list of identified phospholipid and sphingolipid species. Table S3. The full list of lipid species shown in Figure 2d.

## Abbreviations

The following abbreviations are used in this manuscript:

ALT	Alanine aminotransferase
AST	Aspartate aminotransferase
CDP	Cytidine diphosphate
Cers2	Ceramide synthase 2
CoQ	Coenzyme Q
MAS	MASLD activity score
MASH	Metabolic dysfunction-associated steatohepatitis
MASLD	Metabolic dysfunction-associated steatotic liver disease
MCD	Methionine choline-deficient
MCS	Methionine choline-sufficient
PA	Phosphatidic acid
PC	Phosphatidylcholine
PE	Phosphatidylethanolamine
PEMT	Phosphatidylethanolamine N-methyltransferase
PG	Phosphatidylglycerol
PI	Phosphatidylinositol
PS	Phosphatidylserine
Smpd1	Sphingomyelin phosphodiesterase 1
sPLS-DA	Sparse partial least squares discriminant analysis
