# Supplementary material for: Coenzyme Q10 Supplementation Modulates Hepatic Lipidomic Alterations and Attenuates Metabolic Dysfunction-Associated Steatohepatitis in Mice
Source: Nutrients. 2026 Feb 11;18(4):588. doi: 10.3390/nu18040588 (PMC12942857; doi:10.3390/nu18040588)
Supplement: Supplementary file 1 [file nutrients-18-00588-s001.zip › nutrients-4097310-supplementary.pdf]

Supplementary Material

Table S1. Summary of primer sequences

Table S2. Complete list of identified phospholipid and sphingolipid species

Table S3. The full list of lipid species shown in Figure 2d

Table S1. Summary of primer sequences

	Forward	Reverse
Pemt	GAAGCTGAGCAGAGCCTTCG	GACAGCACAAACACGAATCCC
Pcyt1α	AGAATGAGCGCTATGACGCC	CGCTCCCTGCAGAAGAATAGG
Pcyt2	CTATGACATGGTGCATTATGGC	CTGTACCATCTTGTACCTCTCC
Pisd	TCAGTCAGAGAAGCAGCCAGGAC	CAGGAGGTAGTGGAGGATGGTCAG
Cers2	TCTACTGGTCCCTGCTCTTCA	CCAGGAGAAGCAGAGGAGAAT
Smpd1	GGCTCTTGATCAACTCCACAG	GACAATGTCCTGGAGGGATGT

Table S2. Complete list of identified phospholipid and sphingolipid species

Subclass	Name
Ceramide (Cer)	CerP(d18:1/26:0)
	Cer(t18:1/24:0)
	Cer(t18:1/23:0)
	Cer(t18:1/23:0(2OH))
	Cer(t18:1/17:0(2OH))
	Cer(t18:0/23:0)
	Cer(t18:0/17:0(2OH))
	Cer(d18:2/25:0)
	Cer(d18:2/23:0)
	Cer(d18:2/22:0)
	Cer(d18:2/20:1)
	Cer(d18:2/20:0)
	Cer(d18:2/16:0)
	Cer(d18:1/26:1)
	Cer(d18:1/24:2)
	Cer(d18:1/24:0)
	Cer(d18:1/23:0)
	Cer(d18:1/22:0)
	Cer(d18:1/20:0)
	Cer(d18:1/18:1)
	Cer(d18:1/18:0)
	Cer(d18:1/17:0)
	Cer(d18:1/16:0)
	Cer(d18:0/h24:0)
	Cer(d18:0/h17:0)
	Cer(d18:0/24:1)
	Cer(d18:0/24:0)
	Cer(d18:0/23:0)
	Cer(d18:0/22:0)
	Cer(d18:0/18:0)
	Cer(d18:0/16:0)
	Cer(d18:0/14:0)
	Cer(d17:1/24:1)
	Cer(d17:1/22:0)
	Cer(d16:1/17:0)
	1-O-pentacosanoyl-Cer(d18:1/16:0)
	1-O-eicosanoyl-Cer(d18:1/16:0)
	1-O-cerotoyl-Cer(d18:1/16:0)

Subclass	Name
Phosphatidic Acid (PA)	PA(P-18:0/22:6)
	PA(P-16:0/22:6)
	PA(P-16:0/16:1)
	PA(O-20:0/22:6)
	PA(O-20:0/22:1)
	PA(O-20:0/22:0)
	PA(O-20:0/20:5)
	PA(O-20:0/20:3)
	PA(O-20:0/18:0)
	PA(O-20:0/17:2)
	PA(O-20:0/16:0)
	PA(O-18:0/22:6)
	PA(O-18:0/22:1)
	PA(O-18:0/20:5)
	PA(O-18:0/20:3)
	PA(O-18:0/18:4)
	PA(O-18:0/18:3)
	PA(O-18:0/15:0)
	PA(O-16:0/22:6)
	PA(O-16:0/20:2)
	PA(O-16:0/20:1)
	PA(O-16:0/19:1)
	PA(O-16:0/18:4)
	PA(O-16:0/18:3)
	PA(O-16:0/18:2)
	PA(O-16:0/16:1)
	PA(22:2/22:6)
	PA(22:1/22:6)
	PA(22:1/22:2)
	PA(22:0/22:4)
	PA(20:2/22:6)
	PA(20:2/22:4)
	PA(20:1/22:6)
	PA(20:1/22:2)
	PA(19:0/22:4)
	PA(18:4/22:4)
	PA(18:2/22:2)
	PA(18:2/20:5)
	PA(18:1/22:2)
	PA(18:0/22:2)
	PA(18:0/18:2)

Subclass	Name
Phosphatidic Acid (PA)	PA(18:0/18:1)
	PA(17:1/22:6)
	PA(17:0/22:0)
	PA(17:0/20:4)
	PA(16:1/22:4)
	PA(16:1/22:2)
	PA(16:0/22:6)
	PA(16:0/18:2)
	PA(16:0/18:1)
	PA(16:0/16:0)
	PA(15:1/22:4)
	PA(15:1/22:2)
	PA(15:0/20:3)
	LPA P-20:0
	LPA 22:6
	LPA 22:4
	LPA 22:2
	LPA 22:1
	LPA 20:2
	LPA 20:1
	LPA 18:1
	LPA 18:0
	LPA 16:1
Phosphatidyl choline (PC)	PC(P-20:0/22:4)
	PC(P-20:0/19:1)
	PC(P-16:0/15:1)
	PC(O-20:0/22:6)
	PC(O-20:0/22:4)
	PC(O-20:0/20:3)
	PC(O-20:0/17:2)
	PC(O-18:0/22:6)
	PC(O-18:0/22:4)
	PC(O-18:0/22:1)
	PC(O-17:0/22:0)
	PC(O-16:0/22:2)
	PC(O-16:0/20:5)
	PC(O-16:0/20:4)
	PC(O-16:0/20:2)
	PC(O-16:0/20:1)
	PC(O-16:0/18:3)
	PC(O-16:0/18:0)

Table S2. (continued)

Subclass	Name
Phosphatidyl choline (PC)	PC(O-16:0/17:2)
	PC(O-16:0/17:1)
	PC(O-15:0/20:4)
	PC(O-14:0/18:1)
	PC(O-14:0/16:1)
	PC(O-14:0/16:0)
	PC O-41:3
	PC O-40:8
	PC O-40:7
	PC O-40:5
	PC O-38:7
	PC O-38:6
	PC O-38:5
	PC O-38:4
	PC O-37:4
	PC O-36:5
	PC O-36:3
	PC O-36:2
	PC O-36:0
	PC O-34:2
	PC O-34:1
	PC O-34:0
	PC O-32:1
	PC O-32:0
	PC 22:6_22:6
	PC 22:4_22:6
	PC 22:1_22:4
	PC 22:1_22:2
	PC 22:0_22:6
	PC 22:0_22:4
	PC 22:0_18:2
	PC 20:5_22:6
	PC 20:4_22:6
	PC 20:4_22:5
	PC 20:4_20:4
	PC 20:3_22:6
	PC 20:3_18:4
	PC 20:2_22:6
	PC 20:1_22:6
	PC 20:1_22:2
	PC 20:0_22:6

Subclass	Name
Phosphatidyl choline (PC)	PC 20:0_22:5
	PC 20:0_22:4
	PC 20:0_20:4
	PC 20:0_20:3
	PC 20:0_20:2
	PC 20:0_18:2
	PC 19:1_22:4
	PC 19:0_22:6
	PC 19:0_18:2
	PC 19:0_18:1
	PC 18:4_20:5
	PC 18:3_22:6
	PC 18:2_20:3
	PC 18:2_18:2
	PC 18:1_22:6
	PC 18:1_20:4
	PC 18:1_18:2
	PC 18:0_22:6
	PC 18:0_22:5
	PC 18:0_22:3
	PC 18:0_20:4
	PC 18:0_20:3
	PC 18:0_18:1
	PC 18:0_18:0
	PC 17:2_22:6
	PC 17:1_22:6
	PC 17:1_22:2
	PC 17:1_20:4
	PC 17:1_18:2
	PC 17:0_22:6
	PC 17:0_22:4
	PC 17:0_22:2
	PC 17:0_22:1
	PC 17:0_20:4
	PC 17:0_18:2
	PC 17:0_18:1
	PC 16:1_22:6
	PC 16:1_20:4
	PC 16:1_18:2
	PC 16:0_24:1
	PC 16:0_22:6

Subclass	Name
Phosphatidyl choline (PC)	PC 16:0_20:5
	PC 16:0_20:4
	PC 16:0_20:3
	PC 16:0_18:3
	PC 16:0_18:2
	PC 16:0_18:1
	PC 16:0_18:0
	PC 16:0_17:2
	PC 16:0_16:1
	PC 16:0_16:0
	PC 15:1_22:4
	PC 15:1_22:2
	PC 15:0_22:6
	PC 15:0_22:2
	PC 15:0_20:5
	PC 15:0_20:4
	PC 15:0_20:3
	PC 15:0_18:1
	PC 15:0_16:0
	PC 14:1_22:6
	PC 14:0_22:6
	PC 14:0_20:5
	PC 14:0_20:4
	PC 14:0_18:3
	PC 14:0_18:2
	PC 14:0_16:0
	LPC P-15:0
	LPC O-20:0
	LPC O-18:2
	LPC O-18:1
	LPC O-18:0
	LPC O-17:0
	LPC O-16:1
	LPC O-16:0
	LPC O-15:0
	LPC 24:0
	LPC 22:6
	LPC 22:5
	LPC 22:4
	LPC 22:0
	LPC 20:5

Table S2. (continued)

Subclass	Name
Phosphatidyl choline (PC)	LPC 20:4
	LPC 20:3
	LPC 20:2
	LPC 20:1
	LPC 20:0
	LPC 19:2
	LPC 19:0
	LPC 18:3
	LPC 18:2
	LPC 18:1
	LPC 18:0
	LPC 17:1
	LPC 17:0
	LPC 16:1
	LPC 16:0
	LPC 15:0
	LPC 14:1
	LPC 14:0
Phosphatidyl ethanolamine (PE)	PE(P-20:0/22:4)
	PE(P-18:1/22:6)
	PE(P-18:0/22:6)
	PE(P-16:0/20:5)
	PE O-22:6_18:2
	PE O-22:2_20:4
	PE O-20:1_22:6
	PE O-20:1_20:4
	PE O-18:2_24:4
	PE O-18:2_20:4
	PE O-18:1_22:6
	PE O-18:1_22:5
	PE O-18:1_22:4
	PE O-18:1_20:4
	PE O-18:1_18:2
	PE O-16:2_22:1
	PE O-16:1_22:6
	PE O-16:1_22:5
	PE O-16:1_22:4
	PE O-16:1_20:4
	PE O-16:1_18:1
	PE O-16:0_22:5
	PE O-16:0_20:4

Subclass	Name
Phosphatidyl ethanolamine (PE)	PE O-16:0_18:3
	PE 22:6_22:6
	PE 22:6_20:5
	PE 20:4_22:6
	PE 20:4_20:5
	PE 20:4_20:4
	PE 20:3_22:6
	PE 20:2_22:4
	PE 20:2_20:2
	PE 20:0_18:1
	PE 19:1_19:1
	PE 18:4_22:6
	PE 18:2_20:4
	PE 18:1_22:6
	PE 18:1_20:4
	PE 18:0_22:6(14OH)
	PE 18:0_22:6
	PE 18:0_22:5
	PE 18:0_20:4(12OH)
	PE 18:0_20:4
	PE 18:0_20:3
	PE 18:0_18:2
	PE 18:0_18:1
	PE 18:0_16:1
	PE 16:1_22:6
	PE 16:1_20:4
	PE 16:0_22:5
	PE 16:0_20:4
	PE 16:0_18:2
	PE 14:0_22:6
	NAPE(18:1/16:1/18:0)
	LPE O-19:2
	LPE 22:6
	LPE 22:5
	LPE 22:4
	LPE 20:5
	LPE 20:4
	LPE 20:3
	LPE 20:2
	LPE 18:3
	LPE 18:2

Subclass	Name
	LPE 18:1
	LPE 18:0
	LPE 16:1
	LPE 16:0
	LPE 14:0
Phosphatidyl glycerol (PG)	PG(P-20:0/22:2)
	PG(P-18:0/22:6)
	PG(P-16:0/20:5)
	PG(O-20:0/14:1)
	PG(O-18:0/22:6)
	PG(O-18:0/22:1)
	PG(O-18:0/20:5)
	PG(O-18:0/18:4)
	PG(O-16:0/22:1)
	PG(O-16:0/20:5)
	PG(O-16:0/14:1)
	PG 22:6_20:5
	PG 20:4_22:6
	PG 20:2_22:6
	PG 20:2_22:4
	PG 19:0_22:1
	PG 19:0_22:0
	PG 18:4_22:6
	PG 18:3_22:6
	PG 18:3_20:5
	PG 18:2_22:6
	PG 18:2_22:2
	PG 18:2_20:5
	PG 18:1_22:6
	PG 18:1_22:4
	PG 18:1_20:4
	PG 18:1_18:2
	PG 18:1_18:1
	PG 18:0_20:4
	PG 18:0_18:1
	PG 18:0_18:0
	PG 18:0_16:1
	PG 17:0_22:1
	PG 17:0_22:0
	PG 16:1_22:6
	PG 16:1_22:4

Table S2. (continued)

Subclass	Name
	PG 16:1_22:2
	PG 16:0_22:6
	PG 16:0_22:2
	PG 16:0_22:1
	PG 16:0_20:4
	PG 16:0_18:3
	PG 16:0_18:1
	PG 15:0_22:1
	PG 15:0_22:0
	PG 14:1_22:2
	LPG 22:1
	LPG 22:0
	LPG 20:1
	LPG 19:1
	LPG 18:2
	LPG 18:0
Phosphatidyl inositol (PI)	PI(P-18:0/17:2)
	PI(O-20:0/14:0)
	PI(O-16:0/18:3)
	PI(18:4/22:4)
	PI(18:3/22:6)
	PI(18:2/20:5)
	PI(18:1/22:6)
	PI(18:1/22:4)
	PI(18:0/22:2)
	PI(18:0/20:4)
	PI(18:0/18:0)
	PI(17:0/14:1)
	PI(16:1/22:4)
	PI(16:1/22:2)
	PI(16:0/22:2)
	PI(16:0/18:1)
	PI(15:0/20:5)
	PI(14:1/22:2)
	PI(14:0/22:2)
	PI(14:0/22:1)
	PI 40:6
	PI 38:6
	PI 38:5
	PI 36:3
	PI 36:2
	PI 34:2

Subclass	Name
Phosphatidyl serine (PS)	PS(P-20:0/22:2)
	PS(P-16:0/20:5)
	PS(P-16:0/18:4)
	PS(P-16:0/16:1)
	PS(O-20:0/22:6)
	PS(O-20:0/22:2)
	PS(O-20:0/20:0)
	PS(O-20:0/18:0)
	PS(O-20:0/16:0)
	PS(O-18:0/22:6)
	PS(O-18:0/22:4)
	PS(O-18:0/20:3)
	PS(O-18:0/19:1)
	PS(O-18:0/18:3)
	PS(O-18:0/17:2)
	PS(O-18:0/17:0)
	PS(O-18:0/14:0)
	PS(O-16:0/22:6)
	PS(O-16:0/22:4)
	PS(O-16:0/20:5)
	PS(O-16:0/20:2)
	PS(O-16:0/19:1)
	PS(O-16:0/18:3)
	PS(O-16:0/17:1)
	PS(O-16:0/15:1)
	PS 22:2_22:6
	PS 22:2_22:4
	PS 20:3_22:6
	PS 20:2_22:6
	PS 20:1_22:6
	PS 20:1_22:4
	PS 20:1_22:2
	PS 20:0_22:4
	PS 18:2_18:2
	PS 18:1_22:6
	PS 18:1_22:4
	PS 18:1_22:2
	PS 18:0_22:2
	PS 18:0_20:4
	PS 17:1_22:4
	PS 16:0_22:6
	PS 16:0_22:2

Subclass	Name
	PS 16:0_22:1
	PS 16:0_20:4
	PS 16:0_18:1
	PS 16:0_16:0
	PS 15:1_22:2
	PS 15:0_22:2
	PS 14:1_14:1
	LPS 22:6
	LPS 22:2
	LPS 22:1
	LPS 19:1
	LPS 14:1
Glyco-sphingo lipid	LacCer(d18:1/24:1)
	LacCer(d18:1/24:0)
	LacCer(d18:1/22:0)
	LacCer(d18:1/20:0)
	HexCer(d18:1/24:3)
	HexCer(d18:1/24:1)
	HexCer(d18:1/24:0)
	HexCer(d18:1/23:0)
	HexCer(d18:1/22:0)
	HexCer(d18:1/16:0)
	HexCer 43:3;O3
	HexCer 42:1;O2
	HexCer 41:3;O3
	HexCer 40:3;O3
	HexCer 35:3;O3
	HexCer 35:2;O4
	HexCer 35:1;O4
	Hex2Cer(d18:1/24:2)
	GlcCer(d18:2/21:0 (2OH))
	GlcCer(d18:2/20:1)
	GlcCer(d18:2/20:0)
	GlcCer(d18:2/17:0 (2OH))
	GlcCer(d18:2/16:0 (2OH))
	GlcCer(d18:1/26:0)
	GlcCer(d18:1/22:0 (2OH))
	GlcCer(d18:0/22:0)

Table S2. (continued)

Subclass	Name
	GlcCer(d18:0/18:0)
	GlcCer(d16:1/23:0)
	Gb3(d18:1/24:1)
	Gb3(d18:1/22:0)
	asialo-GM2(d18:1/24:1)
	asialo-GM2(d18:1/24:0)
	asialo-GM2(d18:1/22:0)
Sphingomyelin (SM)	SM(t42:1)
	SM(t41:0)
	SM(t40:0)
	SM(t34:1)
	SM(d18:1/25:0)
	SM(d18:1/24:3)
	SM(d18:1/24:2)
	SM(d18:1/24:0)
	SM(d18:1/23:0)
	SM(d18:1/22:2)
	SM(d18:1/22:1)
	SM(d18:1/22:0)
	SM(d18:1/21:0)
	SM(d18:1/20:1)
	SM(d18:1/20:0)
	SM(d18:1/19:0)
	SM(d18:1/18:1)
	SM(d18:1/18:0)
	SM(d18:1/17:0)
	SM(d18:1/16:1)
	SM(d18:1/16:0)
	SM(d18:1/16:0(2OH))
	SM(d18:1/15:0)
	SM(d18:1/14:0)
	SM(d18:0/23:0)
	SM(d18:0/22:0)
	SM(d18:0/20:0)
	SM(d18:0/18:0)
	SM(d18:0/16:0)
	SM(d17:1/26:1)
	SM(d16:1/23:0)

Table S3. The full list of lipid species shown in Figure 2d

PA (O-16:0/19:1)	PA (16:0/16:0)
PA (O-18:0/18:3)	PA (O-20:0/20:3)
PE (O-16:1_18:1)	PA (O-18:0/20:5)
PI (14:1/22:2)	PE (O-16:1_22:5)
PA (O-20:0/22:6)	PG (16:1_22:4)
PG (16:0_22:1)	PE (18:0_22:5)
PG (18:0_18:0)	PE (18:0_18:2)
PE (O-16:1_20:4)	PE (20:2_20:2)
PE (O-20:1_20:4)	PE (O-16:1_22:4)
PE (P-20:0/22:4)	PE (O-18:2_20:4)
PE (O-22:6_18:2)	PG (18:1_18:1)
PE (P-18:1/22:6)	PG (20:4_22:6)
PA (O-16:0/16:1)	PA (22:1/22:6)
PA (O-20:0/18:0)	PE (O-18:2_24:4)
PA (O-20:0/20:5)	PE (O-18:1_22:6)
PS (O-20:0/16:0)	PG (16:0_22:6)
PE (O-18:1_22:5)	PA (17:1/22:6)
PE (O-18:1_22:4)	PA (17:0/20:4)
PE (16:1_22:6)	LPC (O-20:0)
PA (18:4/22:4)	PC (19:0_22:6)
PE (18:1_20:4)	PC (18:1_18:2)
PE (16:1_20:4)	LPC (16:0)
PC (18:2_20:3)	LPC (18:1)
PA (18:2/20:5)	LPG (22:1)
PE (14:0_22:6)	PC (22:1_22:2)
PE (O-20:1_22:6)	LPC (15:0)
PA (16:0/18:2)	PC (16:0_18:2)
PA (18:0/22:2)	PC (18:1_20:4)
PA (20:1/22:6)	PC (18:3_22:6)
PA (P-18:0/22:6)	PS (P-20:0/22:2)
PS (O-20:0/20:0)	LPC (O-16:0)
PE (18:0_22:6)	LPC (17:0)
